# The different interactions of *Colletotrichum gloeosporioides* with two strawberry varieties and the involvement of salicylic acid

**DOI:** 10.1038/hortres.2016.7

**Published:** 2016-03-16

**Authors:** Qing-Yu Zhang, Li-Qing Zhang, Li-Li Song, Ke Duan, Na Li, Yan-Xiu Wang, Qing-Hua Gao

**Affiliations:** 1 Shanghai Key Laboratory of Protected Horticultural Technology, Forestry and Fruit Tree Research Institute, Shanghai Academy of Agricultural Sciences (SAAS), Shanghai 201403, China; 2 College of Landscape Architecture and Arts, Northwest A&F University, Yangling, Shanxi 712100, China; 3 College of Agricultural Sciences, Gansu Agricultural University, Lanzhou 730000, China; 4 School of Life Science, Taizhou University, Taizhou 318000, China

## Abstract

The disease symptoms recognized as ‘Anthracnose’ are caused by *Colletotrichum* spp. and lead to large-scale strawberry (*Fragaria*×*ananassa* Duchesne) losses worldwide in terms of both quality and production. Little is known regarding the mechanisms underlying the genetic variations in the strawberry–*Colletotrichum* spp. interaction. In this work, *Colletotrichum gloeosporioides* (*C. gloeosporioides*) infection was characterized in two varieties exhibiting different susceptibilities, and the involvement of salicylic acid (SA) was examined. Light microscopic observation showed that *C. gloeosporioides* conidia germinated earlier and faster on the leaf surface of the susceptible cultivar compared with the less-susceptible cultivar. Several *PR* genes were differentially expressed, with higher-amplitude changes observed in the less-susceptible cultivar. The less-susceptible cultivar contained a higher level of basal SA, and the SA levels increased rapidly upon infection, followed by a sharp decrease before the necrotrophic phase. External SA pretreatment reduced susceptibility and elevated the internal SA levels in both varieties, which were sharply reduced in the susceptible cultivar upon inoculation. The less-susceptible cultivar also displayed a more sensitive and marked increase in the transcripts of *NB-LRR* genes to *C. gloeosporioides*, and SA pretreatment differentially induced transcript accumulation in the two varieties during infection. Furthermore, SA directly inhibited the germination of *C. gloeosporioides* conidia; *NB-LRR* transcript accumulation in response to SA pretreatment was both dose- and cultivar-dependent. The results demonstrate that the less-susceptible cultivar showed reduced conidia germination. The contribution of SA might involve microbial isolate-specific sensitivity to SA, cultivar/tissue-specific SA homeostasis and signaling, and the sensitivity of *R* genes and the related defense network to SA and pathogens.

## Introduction

Anthracnose, which is caused by the hemibiotroph *Colletotrichum* spp., is one of the most destructive diseases of cultivated strawberry (*Fragaria×ananassa* Duchesne) worldwide.^1^ It occurs perennially from the nursery to the field and causes severe production losses in more than 45% of all strawberry fields in China. Infection by several species of *Colletotrichum* spp. causes strawberry anthracnose, with symptoms such as irregular and black leaf lesions, crown rot, flower blight and fruit rot.^2^ In China, *Colletotrichum gloeosporioides* (*C. gloeosporioides*) has been defined as the major causal agent.^3^ Strawberry susceptibility to anthracnose has been suggested to be polygenic and quantitatively inherited. To date, there are no cultivars that exhibit complete resistance to *Colletotrichum* spp., thus aggravating its deleterious effects on strawberry production.^4,5^

Plants have evolved two strategies to detect pathogens.^4,6^ The first class of perception involves the recognition of conserved microbial elicitors called pathogen-associated molecular patterns (PAMPs) by receptor proteins called pattern recognition receptors (PRRs).^6^ The stimulation of PRRs leads to PAMP-triggered immunity (PTI). The second class of perception involves gene-for-gene-type interactions by which plant extracellular receptors recognize pathogen virulence molecule effectors, resulting in effector-triggered immunity (ETI).^6^ Activation of either PTI or ETI could induce systemic acquired resistance (SAR), which is a form of long-lasting immunity to a broad spectrum of pathogens.^7,8^

The phenolic compound salicylic acid (SA) has an extensive signaling role in plants, particularly in pathogen defense. SA and related immune responses are important parts of both PTI and ETI, and are required for their activation.^9–11^ SA accumulation and signaling are critical for the disease resistance conferred by SAR, especially for the activation of SAR.^12–14^ SA perception and the subsequent transcriptional activation of defense genes are continuous events. NPR1, which is the master regulator controlling multiple immune responses, including SAR, was identified as a direct SA receptor.^15^ The paralogs NPR3 and NPR4 were identified as low- and high-affinity receptors for SA, respectively.^16^ Currently, it is believed that the NPR1-dependent pathway functions additively with the NPR1-independent pathway in SA signaling.^14^

Many proteins functioning upstream of SA in PTI as well as in ETI have been characterized.^17^ ETI is often initiated by a subset of resistance (*R*) genes, and SA lies downstream of these genes. NB-LRR proteins harboring a nucleotide binding site and leucine-rich repeat domains are of the largest class of known R proteins.^18^ Some NB-LRR proteins act as the genetically defined ‘effector-sensor,’ and others are required for the sensor function, the latter of which is not implicated in effector perception *per se*, and are named ‘helper NB-LRRs.^19^’ A gain-of-function mutation in *Arabidopsis* TNL-type R protein SNC1 led to constitutively activated downstream defense responses, such as the accumulation of SA and the constitutive expression of pathogenesis-related (*PR*) genes.^20^ Three *Arabidopsis* CC-NB-LRR proteins were identified as helper NB-LRRs in ETI, which also function in basal defense via the regulation of SA accumulation and subsequent activation of SA-dependent responses.^21^ However, few reports on the directional or feedback regulation of SA on the expression of receptor or helper *NB-LRR* genes are available.

Several studies have correlated the SA levels with increased resistance to *Colletotrichum* spp. or other fungal pathogens in strawberry.^22–26^ Treatment with SA or its analogue benzothiadiazole S-methyl ester (BTH) effectively reduced disease severity or fungal decay in strawberry.^27–29^ Many strawberry genes have been reported to be responsive to *C. acutatum* or *C. fragariae* infection.^30–33^ However, those receptors and the signaling pathways involved in the interactions between strawberry and microbial pathogens remain largely obscure.^5^ It is well known that variation in host resistance is frequently controlled by the segregation of a single resistance (R) gene, and cultivar resistance is tightly associated with gene-for-gene recognition.^34,35^ To date, substantial differences in susceptibility to *C. acutatum* or *C. fragariae* among strawberry cultivars have been reported^30^, but few reports have correlated variations in *R* gene expression with cultivar susceptibility in strawberry.

Previously, through the genome-wide isolation and characterization of *NB-LRR* genes in woodland strawberry (*F. vesca*), we have identified a set of *NB-LRR* genes displaying ecotype-specific responses to *C. gloeosporioides* inoculation.^36^ Some members of these genes might have pathogenesis-related response implications in strawberry. However, the mechanisms underlying the genetic variation in the cultivated strawberry–*Colletotrichum* spp. interaction are largely obscure. In particular, the essential difference between the interactions of *C. gloeosporioides* with susceptible and less-susceptible varieties remains unknown. Is the SA-related defense similarly activated in strawberry varieties with different susceptibilities? What are the potential factors that restrict the contribution of SA in strawberry resistance to *C. gloeosporioides*? Could we correlate the variations in *R* gene expression and SA homeostasis with cultivar susceptibility to *C. gloeosporioides* in strawberry? Here we performed a biochemical and molecular dissection of strawberry–*C. gloeosporioides* interactions, with a particular focus on SA involvement and *NB-LRR* transcript accumulation. The present study sheds light on the intricate genetic variations and SA-related defenses in the interaction between strawberry and its anthracnose pathogen.

## Materials and methods

### Plant materials and growth conditions

Two strawberry cultivars exhibiting different susceptibility to the fungal pathogen *C. gloeosporioides* were used. The cultivar Jiuxiang (JX) is susceptible to *C. gloeosporioides*, whereas cv. Sweet Charlie (H) is less susceptible in field conditions.^37^ Stolon-derived healthy plants with more than 10 fully expanded compound leaves were obtained from a nursery and rooted in pots with sterilized substrate. Before the experiments, pots were transferred to a growth chamber (Conviron, Adaptis A1000AR, Winnipeg, Canada) for 6 weeks and fertilized with 1× Hoagland nutrient solution every week. The conditions in the growth chamber were set as follows: 12-h-light/12-h-dark cycle, 125 μmol m^−2^ s^−1^ photo flux density, 70% relative humidity (RH), and a constant temperature of 25 °C.

For *C. gloeosporioides* inoculation, plants were sprayed to run-off with conidia solution (~10^6^ conidia per mL in sterile distilled water with 0.05% Tween-20), and RH was adjusted to 90±5% for the first 2 days. For exogenous SA treatment, plants were sprayed to run-off with 0, 20, 100 and 500 μM SA (Alfa Aesar, cat. no. A12253, Johnson Matthey Catalog Company, Inc., Ward Hill, MA, USA) in sterile water with 0.05% Tween-20. Plants were maintained in the chamber for 4 days and were then inoculated with *C. gloeosporioides* as mentioned above. The sixth tri-foliate leaves were harvested at 0 (mock treatment with water containing Tween-20), 6, 12, 24, 48 and 96 hours post inoculation (hpi). For each treatment, 3 technical replicates from 15 different individual plants were sampled at each time point (five plants for one replicate) and either fast stored at −70 °C (for RNA analysis) or freshly used (for hormone extraction). Experiments were independently performed in two different years.

### Cultivation of *C. gloeosporioides* for inoculation

*C. gloeosporioides* (deposited under acc no. CGMCC3.17371) was locally purified and propagated on fresh potato/dextrose/agar (PDA) medium^38^ with streptomycin (30 mg L^−1^) and ampicillin (100 mg L^−1^). This pathogen was sub-cultured on PDA medium without antibiotics at 28 °C. After a 12-day cultivation when the fungus had proliferated throughout the Petri dishes, spores were taken with a Drigalski spatula and suspended in sterile water. The suspension was filtered through a nylon mesh for hyphae retention, and the spore concentration was determined in a Neubauer counting chamber and adjusted to ~10^6^ conidia per mL for inoculation.

### Antimicrobial effects of SA on *in vitro C. gloeosporioides* growth

To test the *in vitro* effects of SA on *C. gloeosporioides* growth, 5-mm-diameter mycelium plugs were aseptically cut from the advancing margins of 7-day-old single-spore PDA colonies using a sterile cork-borer and then transferred to the center of fresh PDA media with SA at different concentrations (0, 2.5, 5, 7.5,10, 25, 50, 100, 200 and 500 μmol L^−1^). The diameters of the colonies were measured after 6–7 days of incubation at 28 °C. For *C. gloeosporioides* conidia germination, a spore suspension (1×10^4^ spores per mL) was deposited as three 100-μL drops onto a ground microscope slide. A minimum of 100 spores was counted for each drop. SA at different concentrations was supplemented in the spore solution. Spore germination was assessed after a 12-h cultivation at 28 °C. A conidium was recorded as being germinated if one or more germ tubes were visible. An equal volume of solvent was used for mock (0 μmol L^−1^) and SA treatments (100 μL methanol in 100 mL PDA media; 50 μL methanol in 1000 μL spore solution). Three to four technical replicates were used for each SA concentration, and two independent experiments were performed.

### Quantification of endogenous strawberry SA by GC-MS/MS

The levels of free SA in *C. gloeosporioides-*infected JX and SW were measured using a gas chromatography-tandem mass spectrometry (GC-MS/MS) technique at 0, 6, 12, 24, 48, and 96 hpi. Plants treated with distilled water with 0.05% Tween-20 under the same conditions were used as non-infected controls (0 h). The sixth compound leaf blades were excised and immediately homogenized with liquid nitrogen. The pooled sample was dissolved in 1.5 mL 80% methanol, and the net fresh weight (50–80 mg) was determined. Free SA was separated and analyzed by GC-MS/MS, as previously described.^39^ The internal standard for SA was purchased from Cambridge Isotope Laboratories (http://www.isotope.com). SA content is expressed as ng mg^−1^ leaf fresh weight (FW). The sixth leaves from 15 plants (divided into three replicates) were sampled at each time point for variety.

### Observation of *C. gloeosporioides* infection using light microscopy

Light microscopy analyses of *C. gloeosporioides* infection were performed as previously described.^40^ Samples were collected at 6, 12, 24, 48 and 96 h after inoculation. Leaves were decolorized in 0.15% trichloroacetic acid in a 3:1 (v/v) mixture of ethanol and chloroform for 48 h (trichloroacetic acid solution was changed at least three times), rinsed by immersion in lactophenol (Sigma, St Louis, MI, USA) for 1 min and then stained with lactophenol blue solution for 5 min. After staining, the tissues were rinsed (2×2 min) in lactophenol, mounted in 50% fresh glycerol on glass microscope slides and examined under a Nikon E200 microscope. Three leaflets of the first, third and the sixth leaves were sampled from each plant, and five plants were observed at each time point for every variety.

### Quantification of symptom development

For certain cultivar, a total of 30 plants were utilized for this study. Half were pretreated with 20 μmol L^−1^ SA and the remaining plants were untreated before inoculation with *C. gloeosporioides.* Five plants were used for one replicate. From each plant, five leaves chosen at random were scored for symptom development at 2, 4, 7 and 10 days post inoculation (dpi). The severity of symptoms is expressed as disease index and leaf incidence. Leaf lesions were grouped into seven scales according to the size: 1st ⩽1 mm^2^, 2nd=1–4 mm^2^, 3rd=4–9 mm^2^, 4th=9–16 mm^2^, 5th=16–25 mm^2^, 6th=25–36 mm^2^ and 7th ⩾36 mm^2^. Disease index was calculated according to the following formula: [∑(number of lesions at each scale×lesion scale)∕total leaves investigated]×100; leaf incidence=(total leaves showing symptom ∕total leaves investigated)×100.

### Gene expression analysis using qRT-PCR

Isolation of strawberry RNA was performed as previously published.^36^ Synthesis of the first-strand cDNA was accomplished using the PrimeScript RT reagent Kit with gDNA Eraser (Takara, Dalian, China, DRR047A) with minor modifications. The Premix Ex Taq (Perfect Real Time) kit (Takara, Dalian, China, DRR041A) was used for quantitative real-time RT-PCR on an ABI 7300 Real-Time Cycler (Applied Biosystems, Waltham, MA, USA). The fluorescence data collected during the 72 °C step were analyzed with ABI 7300 analysis software. For the qRT-PCR analysis, quantification was based on Ct values. For transcript normalization, *FaRIB413* was used as a reference gene.^41^ A total of five *PR* genes and eight *NB-LRR* genes were analyzed. The sequence information for all RT-PCR primers is given in Supplementary Table S1. Three technical replicates and two biological replicates were conducted. The displayed data are the mean values of two biological replicates, and each value of a biological replicate was derived by averaging the three technical replicates.

### Analysis of statistical significance

For pairwise comparisons between SA treated and untreated data or between cultivars, Student’s *t*-test (two samples assuming unequal variances, in Microsoft Excel 2007) was used. For multiple comparisons of data between cultivar and different time points, a two-way analysis of variance (ANOVA, Statistical Analysis System (SAS) software for Windows 8.0) was performed to analyze the statistical significance. To evaluate the effects of exogenously applied SA on conidia germination, a one-way ANOVA was utilized.

## Results

### The cellular infection process of *C. gloeosporioides*

**Figure 1:**
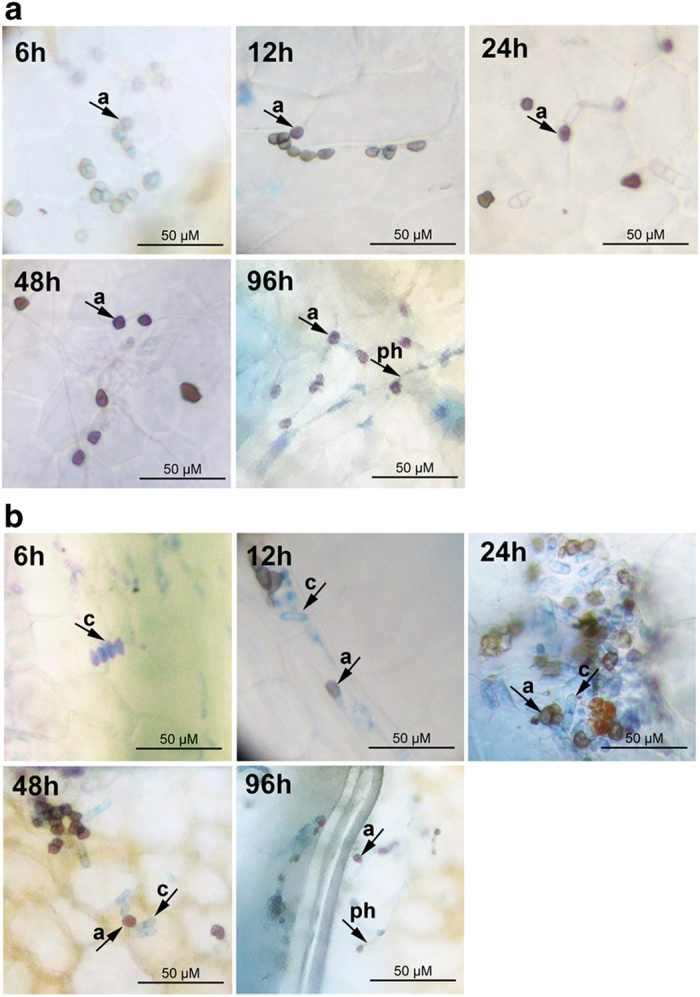
Light microscopic observation of the infection of *C. gloeosporioides* on strawberry leaves. (**a**) Microscopic observation of the infection process on the leaf surface of the susceptible cultivar JX at different time points after inoculation. (**b**) The infection process in the less-susceptible cv. SW. Leaves were observed under a light scene of a Nikon E20 microscope after staining with lactophenol blue for 5 min. Scale bars, 50 μm. *a*, appressorium; *c*, conidium; *ph*, primary hyphae.

Light microscopic observation was performed to reveal the process of cellular infection process of *C. gloeosporioides* on the leaf surfaces of two strawberry cultivars. After staining with lactophenol blue, conidia in uniform long elliptical shape are stained in blue, whereas appressoria in irregular spherical shape are mostly stained in brown. As shown in Figure 1, conidia germinated much faster and earlier on leaves of cv. JX than on those of cv. SW. By 6 hpi, many appressoria were observed on leaves from cv. JX, but none of the conidia was found to germinate in cv. SW. The first detection of the conidium germination in cv. SW was by 12 hpi. By 24 hpi, fewer than half of conidia germinated in cv. SW, whereas nearly all conidia germinated in cv. JX. By 48 hpi, un-germinated conidia could still be found on the leaves of cv. SW. However, the present study did not detect obvious difference in hyphae growth between the cultivars. Primary hyphae were observed at 72 hpi, and the growth traits (density and length) of primary hyphae were similar between the cultivars by 96 hpi (data not shown).

### The expression responses of strawberry *PR* genes

**Figure 2:**
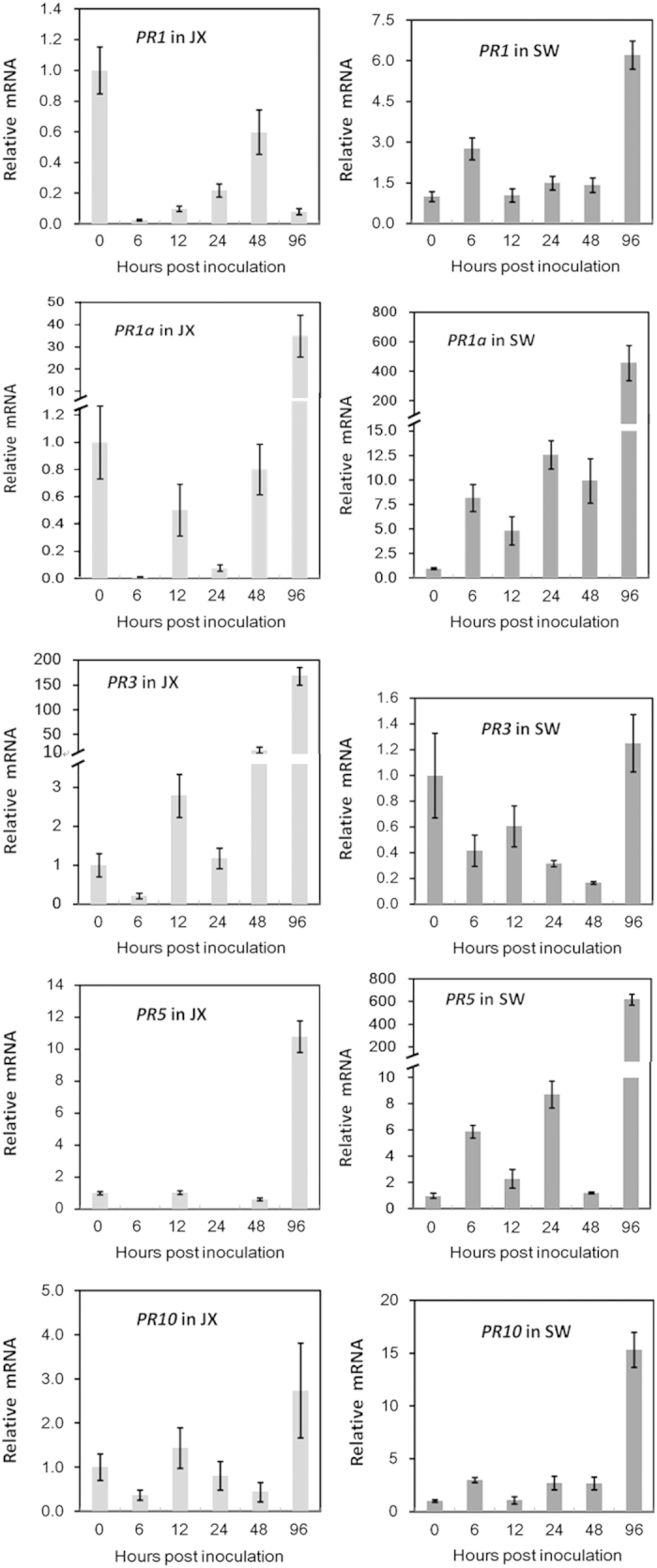
Transcript accumulation of pathogenesis-related genes (*PR*s) during *C. gloeosporioides* infection in two strawberry cultivars. The expression values of all genes were calculated relative to 0 hpi (mock-treated plants, set to 1) in each variety. *FaRIB413* was used for normalization. Bars represent the mean of two independent biological experiments±s.e. Three technical replicates were included for each biological sample.

PR proteins are closely related to the activation of hormone-mediated defense and pathogen resistance. The dynamic mRNA levels of five *PR* genes were evaluated in *C. gloeosporioides-*infected strawberry. All of the *PR* genes tested except *PR3* were inversely regulated by 6 hpi, that is, they were repressed in the susceptible cultivar JX while being induced in the less-susceptible cv. SW (Figure 2). By 96 hpi, this difference in the gene response was maintained for *FaPR1*, whereas *FaPR1a*, *FaPR5* and *FaPR10* were significantly induced in both cultivars. Generally, the changes in *PR* transcripts were more drastic in cv. SW than in cv. JX, especially for *FaPR1a* and *FaPR5*. *FaPR3* was suppressed in both cultivars at 6 hpi. Later, its transcript levels were found to be upregulated in cv. JX but continuously downregulated in cv. SW. By 96 hpi, the transcript level of *FaPR3* was restored to the basal level in cv. SW but was drastically increased in cv. JX.

### The influence of SA pretreatment on strawberry symptom development

The effects of SA on the outcomes of plant–pathogen interactions were evaluated to reveal their potential involvement. Symptoms in strawberry plants pretreated with or without SA were scored at 2, 4, 7 and 10 dpi using a disease severity rating (disease index) and the percentage of leaves showing disease symptoms (leaf incidence). Light lesions coincident with cell necrosis were first observed on leaves at 2 dpi in both cultivars, although it was more noticeable in the cv. JX than in the cv. SW. Pairwise comparisons revealed that there were significant differences in disease severity before 10 dpi between the cultivars (Supplementary Table S2). These observations supported the notion that cv. SW was relatively less susceptible.

**Figure 3:**
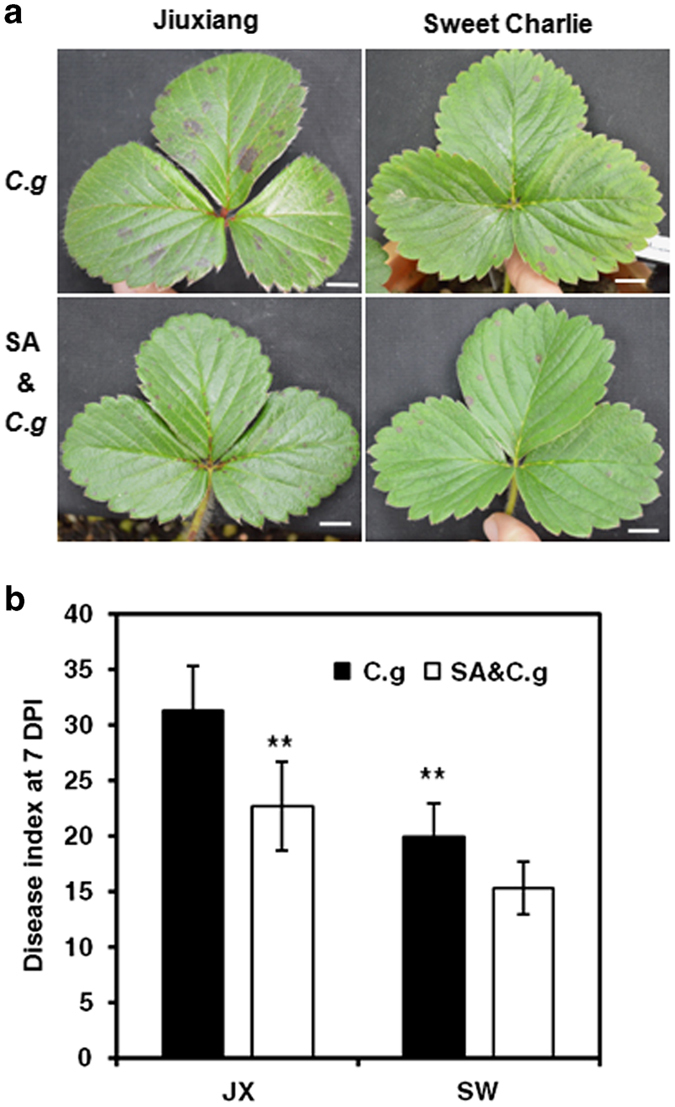
Macroscopic symptoms of *Fragaria ×ananassa* (cvs. JX and SW) leaves inoculated with *C. gloeosporioides*. For salicylic acid (SA) pretreatment, foliar spraying of 20 μM SA was applied 4 days before *C. gloeosporioides* inoculation. (**a**) Macroscopic symptoms at 7 days post inoculation (dpi). Scale bars, 1 cm. (**b**) Disease index at 7 dpi. Significant differences between SA treatments or cultivars were indicated (*t*-test: **P-value <0.01). Error bars represent s.d. (*n*=3).

Foliar spraying with 20 μM SA 4 days before *C. gloeosporioides* inoculation repressed symptom development in both cultivars, which was reflected by smaller and fewer leaf lesions, reduced disease index and decreased leaf incidence (Figure 3). Typical symptoms in a single compound leaf and a whole plant at 7 dpi are shown in Figure 3a and Supplementary Figure S1, respectively. SA pretreatment significantly reduced the average disease index in cv. JX from 31.4 to 22.9 at 7 dpi. However, in cv. SW, this index was not significantly reduced from 20 to 15.3 (Figure 3b). Consistently, SA had a positive influence on reducing strawberry susceptibility to *C. gloeosporioides* infection in both cultivars.

### Free SA accumulation during *C. gloeosporioides* infection

**Figure 4:**
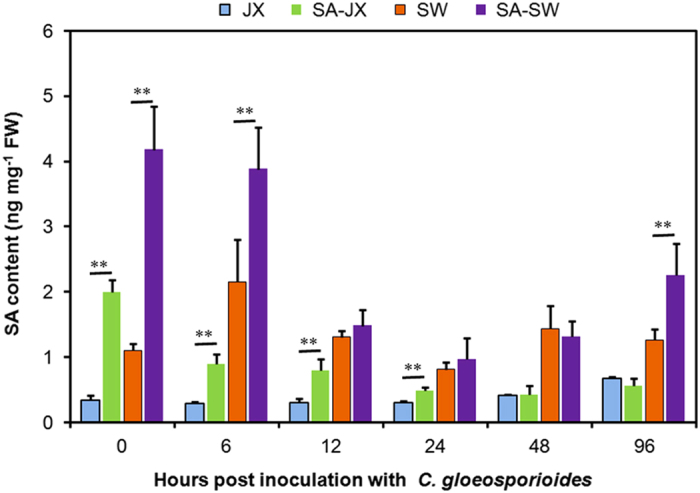
SA content in *Fragaria*×*ananassa* during *C. gloeosporioides* infection. Free SA was determined in the sixth leaves after mock treatment (0 hpi) or *C. gloeosporioides* inoculation in cv. JX (susceptible) or cv. SW (less susceptible) with (green bars, SA pretreated cv. JX; purple bars, SA pretreated cv. SW) or without 20 μM SA pretreatment (blue bars, cv. JX; orange bars, cv. SW). Bars represent the mean values of three independent biological samples±s.d. A paired Student’s *t*-test was performed to analyze the effect of SA pretreatment at every time point; **P<0.01. A paired *t*-test of the significance in the differences between cultivars was found at every time point and is not shown in the figure.

Free SA content was measured in *C. gloeosporioides*-infected or mock-treated strawberry leaves. Under the same controlled conditions, the basal level of free SA in the leaves of cv. JX and cv. SW was 0.33 and 1.11 ng mg^−1^ FW, respectively (Figure 4, 0 hpi). With or without SA pretreatment, the less-susceptible cv. SW contained two-fold or greater free SA than did the susceptible cv. JX at 0 hpi (under the same mock treatment). Indeed, it was found that cv. SW contained significantly higher levels of free SA than did cv. JX at every time point investigated, regardless of exogenous SA pretreatment.

Exogenous SA pretreatment 4 days before inoculation significantly increased the internal free SA content in both varieties at 0 hpi. During infection in cv. JX, the significant increase in internal SA resulted from exogenous SA pretreatment was maintained from 0 hpi until 24 hpi. In cv. SW, the effect of exogenous SA pretreatment was only maintained at 6 and 96 hpi.

Dynamic free SA levels markedly reflected that the two cultivars have different responses to *C. gloeosporioides* infection. As shown in Figure 4 and Table 1, the statistical significance was quite notable at early stages. With no exogenous SA pretreatment, the inoculation induced a transient increase in free SA accumulation at 6 hpi in cv. SW (orange bar), but no significant change was observed in cv. JX (blue bar). Indeed, in cv. JX, the dynamic accumulation of free SA was not significantly changed in the first 48 h post infection, and an obvious increase in SA levels was only observed at 96 hpi. In contrast, the free SA in cv. SW was transiently increased up to 194% by 6 hpi. Later, a significant sharp decrease in SA content was observed in cv. SW by 24 hpi. After that point, SA content was no longer significantly changed in cv. SW, although a slight increase was observed. Under SA pretreated conditions, the inoculation induced a significant sharp decrease in free SA levels in cv. JX (green bar) at 6 hpi, and a similar decrease in SA levels was delayed in cv. SW (purple bar) to 12 hpi. In addition, a mild increase in SA levels occurred at 96 hpi in cv. SW, but not in cv. JX.

### Strawberry *NB-LRR* responses to *C. gloeosporioides* infection and the influence of SA pretreatment

**Figure 5:**
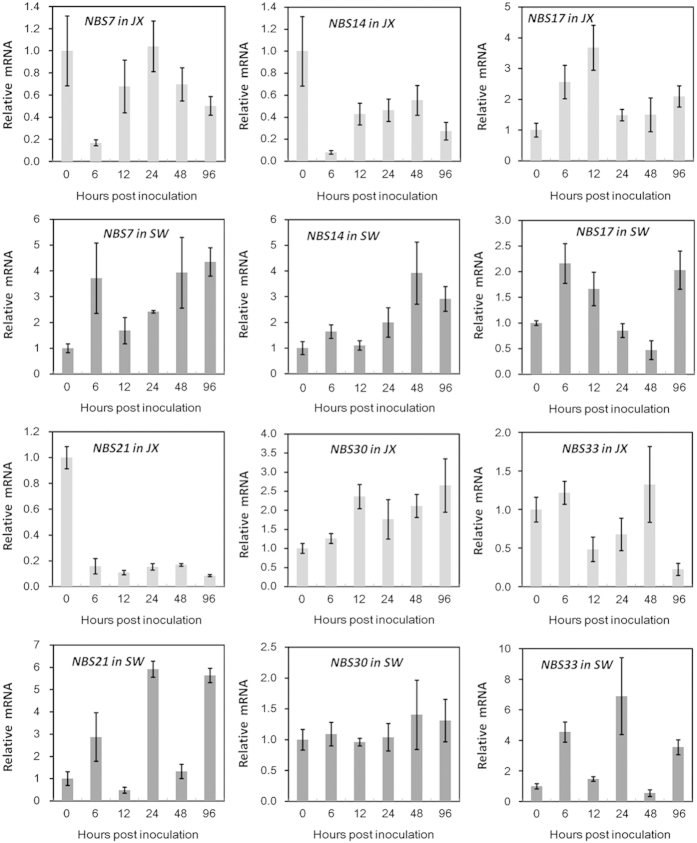
qRT-PCR analysis of *NB-LRR* gene transcript levels in strawberry cvs. JX and SW during *C. gloeosporioides* infection. The expression level of each gene in every cultivar is expressed as the fold change relative to that in mock (0 hpi) treatment. *FaRIB413* was used for normalization. Bars represent the mean of two independent biological experiments±s.e.

It is well known that R proteins encoded by the largest class of *NB-LRR* genes are of great importance for genetic variations in the plant defense response system. To correlate variations in *NB-LRR* genes with strawberry susceptibility, we performed qRT-PCR to better understand their transcript accumulation during *C. gloeosporioides* infection. Over 20 *NB-LRR* genes were examined, of which six members with obviously different changes upon infection between the cultivars were found (Figure 5). In cv. JX, *FaNBS17* and *-30* were transiently and moderately upregulated; the remaining four genes were downregulated upon pathogen infection and were either transiently (*FaNBS7* and -*33*) or continuously (*FaNBS14* and -*21*) repressed. However, in cv. SW, aside from *FaNBS30*, which was not obviously affected, the other five members were clearly upregulated at certain stages during infection. The expression patterns of *FaNBS21* and *-33* were quite similar in cv. SW.

**Figure 6:**
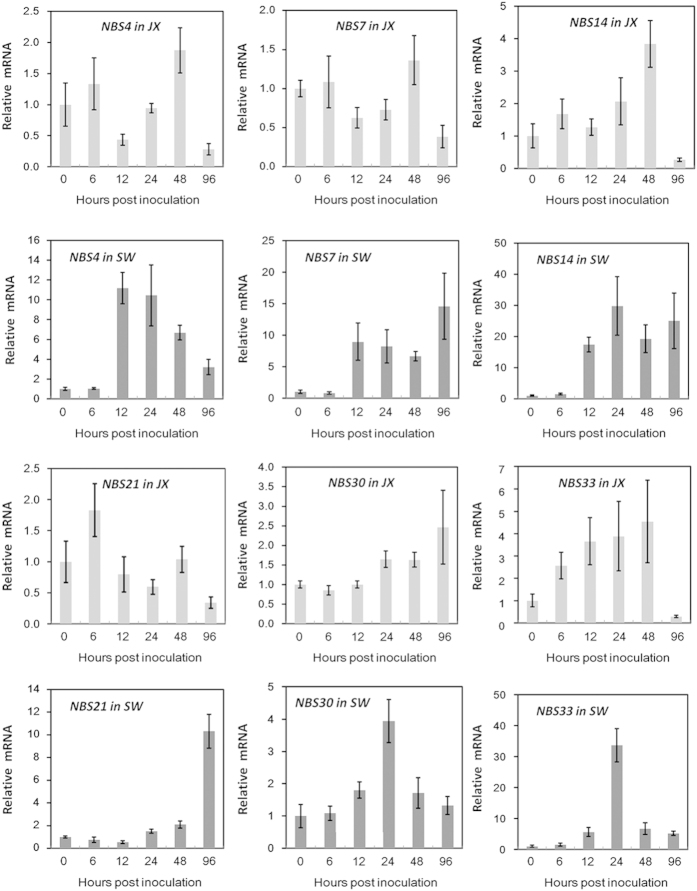
qRT-PCR analysis of *NB-LRR* gene transcript levels in strawberry cvs. JX and SW during *C. gloeosporioides* infection after exogenous SA pretreatment. Foliar spraying of 20 μM SA was applied 4 days before *C. gloeosporioides* inoculation. The expression level of each gene in every cultivar is expressed as the fold change relative to that in mock (0 hpi) for *C. gloeosporioides* inoculation. *FaRIB413* was used for normalization. Bars represent the mean of two independent biological experiments±s.e.

Treatment with SA 4 days before *C. gloeosporioides* inoculation induced clear changes in the transcript accumulation of a set of *FaNBS* genes in both cultivars. *FaNBS17* was the unique member that was not obviously altered by SA pretreatment during *C. gloeosporioides* infection (data not shown). Compared with the lack of SA treatment (Figure 5), SA pretreatment clearly induced all five genes plus *FaNBS4* during *C. gloeosporioides* infection in cv. SW, whereas all but *FaNBS7* were moderately enhanced in cv. JX (Figure 6). For most of these genes, the induction of expression by SA pretreatment during *C. gloeosporioides* infection was more drastic and lasted longer in cv. SW than in cv. JX. In cv. SW, a notable peak in the transcript levels of *FaNBS7* and *-21* was observed by 96 hpi; for *FaNBS4*, -*14*, -*30* and -*33*, their transcripts peaked at ~24 hpi. In contrast in cv. JX, a moderate and transient upregulation was only observed for *NBS14*, -*21* and -*30* at different time points. *FaNBS33* expression was continuously activated in cv. JX during the first 48 hpi. By 96 hpi, compared with the corresponding basal level, the expression of all genes except for *FaNBS30* was no longer enhanced and was even inhibited in cv. JX, which was contrary to that observed in cv. SW.

Together, a set of *R* genes including at least *FaNBS4, -7*, *-14*, -*17*, -*21*, -*30* and *-33* could be involved in the genetic variations in strawberry susceptibility to *C. gloeosporioides*. Exogenous SA pretreatment differentially changed the transcript levels of *NB-LRR* genes in two cultivars. In most instances, SA pretreatment activated or enhanced the expression of *NB-LRR* genes. However, the early expression responses of *FaNBS7*, -*21* and -*33* to *C. gloeosporioides* infection were eliminated by SA pretreatment at 6 hpi in cv. SW.

### Dosage effects of SA on *C. gloeosporioides* viability and strawberry *NB-LRR* gene expression

**Figure 7:**
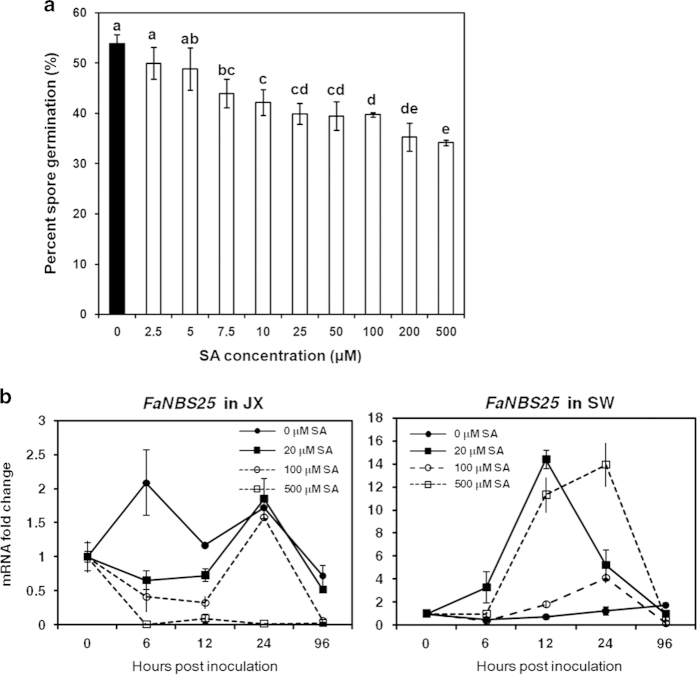
Dosage effects of SA on *in vitro C. gloeosporioides* conidia germination and strawberry *NB-LRR* gene *FaNBS25* transcript accumulation. (**a**) Germination of *C. gloeosporioides* conidia after 12 h at 28 °C in different SA solutions. For mock treatment (0 μM SA), an equal volume of methanol (SA solvent, 5%) was added. Different letters indicate a significant difference between SA concentrations according to the corresponding one-way ANOVA (SAS software for Windows 8.0, *P*<0.05). (**b**) Expression of *FaNBS25* during *C. gloeosporioides* infection in cvs. JX and SW pretreated with SA at different concentrations 4 days before inoculation. *FaNBS25* mRNA is expressed as the fold change relative to that in the mock (0 hpi). Bars represent the mean of two independent biological experiments±s.e.

A survey of the potential influence of SA on fungal viability should be beneficial for understanding its involvement in strawberry–*C. gloeosporioides* interaction. A previous report showed that SA at 5 mM did not inhibit the growth of *Colletotrichum fragariae*.^24^ We examined the effects of SA ranging from 2.5 to 500 μM on *C. gloeosporioides* viability. Hypha growth was not sensitive to SA. In fact, the weak inhibition of 500 μM SA on *C. gloeosporioides* hypha growth was not significant compared with the mock treatment (data not shown). In contrast, a significant suppression of conidia germination was visible for SA concentrations as low as 7.5 μM (Figure 7a). SA at 500 μM reduced the germination rate of *C. gloeosporioides* conidia from 54 to 35%. The inhibitory effect of 100 μM SA on conidia germination was not significantly different from that of 25, 50 or 200 μM SA.

The potential dose-dependent responses of the *NB-LRR* gene to SA were further investigated during *C. gloeosporioides* infection. *FaNBS25* suppression by SA pretreatment in cv. JX was selected for this investigation (Figure 7b). The transcript levels of *FaNBS25* during infection appeared to be inversely affected by SA pretreatment in two cultivars. Pretreatment with 500 μM SA strongly suppressed *FaNBS25* transcript accumulation upon infection in cv. JX, but this treatment markedly enhanced its expression in cv. SW. In cv. JX, the *FaNBS25* transcript level was also suppressed by 20 or 100 μM SA before 12 hpi, followed by a transient increase, as observed in untreated SA inoculation. By 96 hpi, this gene was clearly suppressed in cv. JX for all SA pretreatments. In contrast in cv. SW, pretreatment with SA at 20 or 500 μM clearly enhanced the *FaNBS25* steady-state transcript levels during *C. gloeosporioides* infection. Notably, 100 μM SA showed a relatively weaker induction of *FaNBS25* transcript levels compared with 20 or 500 μM SA. These results highlight the robustness and complexity of the *NB-LRR* transcript response to SA. Clearly, there are not simple linear relationships between exogenously applied SA and pathogen viability or the host resistance gene response.

## Discussion

It has been suggested that there is a continuum of possible plant–pathogen interactions ranging from complete resistance to extreme susceptibility.^42^ However, especially in hemibiotrophic or necrotrophic interactions, incomplete and quantitative disease resistance is very widespread. This is also the case for the interactions between strawberry and *Colletotrichum* spp. To date, no strawberry cultivar or variety has been found to show complete resistance to *Colletotrichum* spp. Here we studied the infection process using two cultivars showing different susceptibility and found that the germination of *C. gloeosporioides* conidia and the formation of appressorium (a structure that enables the pathogen to penetrate the plant cuticle or pores) were much slower in the less-susceptible strawberry cultivar SW. It is likely that cv. SW utilizes unrecognized strategies to block *C. gloeosporioides* infection at the outset. Consistently, by sensing host-specific characteristics and substances, plant-pathogenic fungi germinate only in the presence of a suitable host plant.^43^ Furthermore, in *Colletotrichum* species, conidial germination is triggered by the waxes and ethylene produced by the host plant; contact with a hard surface is a prerequisite for the chemical signal to allow the effective initiation of germination.^44^ SA may be an anti-conidial germination factor because a direct influence of exogenous SA close to physiological concentrations of SA was found on *in vitro C. gloeosporioides* conidia germination. The less-susceptible cultivar did contain higher basal SA, although this does not indicate that such a concentration is the same in the extracellular space, for example, the leaf surface. Clearly, many factors may influence this variation in conidia germination, but we propose that reduced susceptibility versus susceptibility denotes delayed or fewer germinated conidia in the former versus the latter.

SA is critical for plant survival under abiotic and/or biotic stresses because it acts as the central regulator of immunity. Exogenous SA exhibited high potential to control strawberry postharvest losses.^27,45^ The SA analogue BTH induced the expression of several defense genes in strawberry.^46^ Due to the allocation costs and negative effects on plant growth and reproduction, SA-inducible defense and SA homeostasis are strictly regulated in plants.^47^ However, the roles and regulatory mechanisms of SA biosynthesis remain elusive. It was reported that the SA content was greatly affected by strawberry varieties and cultivation conditions, but the endogenous SA content in ripe fruits did not correlate with the fruit susceptibility to powdery mildew caused by the fungus *Sphaerotheca macularis*.^48^ The role of SA in the interactions between the plant host and hemibiotrophs such as *Colletotrichum* spp. might be more complicated than expected, as SA both negatively and positively interacts with many metabolites and signaling molecules.

The present study showed that the less-susceptible strawberry cultivar SW was characterized by a high basal level and a transient increase in SA 1 day after *C. gloeosporioides* infection. Consistently, it was reported that the less-susceptible strawberry cultivar Andana harbors almost double the level of basal SA than the susceptible Camarosa; the susceptible cv. Camarosa increased free SA at 3–5 days post inoculation with *Colletotrichum* spp., which was not observed in cv. Andana.^49^ However, this does not indicate that the endogenous basal SA level in a plant is the main cause of susceptibility versus resistance in strawberry because pathogen infection may induce plant responses regulated by SA, jasmonic acid, ABA, ethylene and even auxin in a complicated manner.^50^ Indeed, we measured the basal SA content in 14 different strawberry germplasms of similar developmental and health status. We found that the basal content of SA does not correlate with germplasm-specific susceptibility in the field (data not shown). The present work revealed that the susceptible cv. JX did not elevate SA content upon *C. gloeosporioides* inoculation, as observed in the less-susceptible cv. SW. In addition, exogenous SA pretreatment elevated the internal levels of SA in strawberry, but a sharp decrease in SA levels was observed in both varieties upon inoculation. SA homeostasis in strawberry is quite complex. However, it is clear that the reduction of the host internal SA level is the main strategy of *C. gloeosporioides* for successful infection. In addition, SA relevant immune responses might be hijacked upon *C. gloeosporioides* infection in the susceptible strawberry. The same *PR* genes behave differently, and the marker gene *PR1*, which is relevant to the SA pathway, shows opposite expression patterns in cultivars with varying susceptibility, suggesting that the SA signaling pathway is not efficiently activated in the susceptible cultivar.

As a chemical inducer of plant resistance, exogenous SA exerts a direct influence on pathogen viability and plant gene expression in a dose-dependent manner. Previously, an *in vitro* growth experiment showed that 5 mM SA did not inhibit the growth of the virulent isolate M11of *C. fragariae*, whereas 50 mM SA manifested a clear inhibitory effect.^24^ The present study revealed that 500 μM SA induced no significant inhibition on the growth of *C. gloeosporioides* hypha but that SA as low as 7.5 μM clearly suppressed the germination of *C. gloeosporioides* spores. These results lead us to hypothesize that the sensitivity of a particular microbial isolate to SA could contribute to the specific interaction between *Colletotrichum* spp. and different cultivars of the same species.

As for gene transcript accumulation studies, the dosage effect of SA is more complicated. Previous studies showed that SA at concentrations as low as 0.1 μM could suppress MeJA-induced *PDF1.2* transcription, although an SA concentration <100 μM had no effect on *PR-1* expression.^51^ This work showed that the dose-dependent effect of SA on resistance gene expression is largely influenced by plant genotypes. In certain cultivars, SA activates the transcript accumulation of one *R* gene only at low concentrations, whereas in another cultivar, SA at the same concentration simply suppresses transcript accumulation of that *R* gene. In plants, SA is believed to work downstream of *NB-LRR* genes in defense responses. Some cases of *NB-LRR*-mediated ETI require the SA-signaling molecule as a downstream mediator of transcriptional output responses.^52^ In addition, three members of the CC-NB-LRR ADR1 protein family were revealed to function in basal defense and in response to a disarmed pathogen via the regulation of SA accumulation and the subsequent activation of SA-dependent responses.^21^ We found that exogenous SA could directly activate or suppress the transcription of *NB-LRR* genes dependent on certain genes and the strawberry genotype (Supplementary Figures S2 and S3). This prompted the hypothesis that the internal SA maintains feedback regulation on its upstream *NB-LRR* receptors in the defense network.

It was revealed that the activation of a set of *NB-LRR* resistance genes to *C. gloeosporioides* is quicker and stronger in the less-susceptible cv. SW than in the susceptible cv. JX. Consistent results have been observed in strawberry cv. Andana (less susceptible) and cv. Camarosa (very susceptible) upon infection by *C. acutatum*.^30^ Moreover, the activation of *NB-LRR* gene transcription by SA pretreatment was stronger and lasted longer in cv. SW than in cv. JX during *C. gloeosporioides* infection. Differences in the expression responses of a set of *NB-LRR* genes might contribute to the genetic variations in susceptibility between the varieties. Their expression patterns are complex, as many inconsistencies in *NB-LRR* homologs have been observed between the octoploid variety and the diploid strawberry *Fragaria vesca*. For example, *FvNBS30* is strongly repressed during infection in the susceptible ecotype HLJ3#,^36^ whereas *FaNBS30* continuously increases after infection in the susceptible cultivar JX (this study). Polyploidization often brings silencing and the biased expression of some gene pairs, which are reciprocal and developmentally regulated, and some silencing events are epigenetically induced during the allopolyploidization process.^53^ Consequently, these inconsistencies might result from the fact that the cultivated strawberry is allocotoploid.

*NB-LRR* genes are of great importance for genetic variations in plant susceptibility. In *Phaseolus vulgaris* ^54^ and *Juglans* spp.,^55^ DNA marker(s) adjacent to *NB-LRR* gene(s) were reported to be correlated with anthracnose resistance. Ectopic expression of the *Medicago truncatula NB-LRR* gene *RCT1* in alfalfa resulted in broad-spectrum anthracnose resistance.^56^ The present study sheds some light on the genetic variations and SA-related defenses in the strawberry–*C. gloeosporioides* interaction and enables us to capture valuable genetic components for future functional confirmation and the development of molecular markers associated with disease resistance. A set of *R* genes that includes at least *FaNBS7*, *-14*, *-21* and *-33* could be involved in strawberry susceptibility to *C. gloeosporioides* and have potential applications for breeding to improve resistance. Ongoing studies in our lab are focusing on the regulatory role of SA-responsive *FaNBS* genes in strawberry defense responses.

It is reasonable to speculate that SA-mediated plant responses including the transcript accumulation of resistance genes are both cultivar- and tissue-dependent. Indeed, strawberry fruit was reported to be more susceptible to *C. acutatum* infection than crown tissue.^30^ Thus, it is possible that the role of the SA-mediated defense network in strawberry susceptibility to the anthracnose pathogen is refined by factors that include, at least, cultivar-/tissue-specific SA homeostasis, the sensitivity of *R* genes to SA and the pathogen (also cultivar-/tissue-specific), and microbial isolate-specific sensitivity to SA.

## Figures and Tables

**Table 1:** Least squares mean values for the effect of cultivar×time points on free SA responses

	*SW-0 hpi*	*SW-6 hpi*	*SW-12 hpi*
*SA untreated*			
JX-0 hpi	0.0005	<0.0001	<0.0001
JX-6 hpi	0.0002	<0.0001	<0.0001
JX-12 hpi	0.0003	<0.0001	<0.0001
			
*SA pretreated*			
JX-0 hpi	<0.0001	<0.0001	0.0731
JX-6 hpi	<0.0001	<0.0001	0.0420
JX-12 hpi	<0.0001	<0.0001	0.0188

Abbreviations: hpi, hours post inoculation; JX, Jiuxiang; SA, salicylic acid; SW, sweet Charlie.

*P*-values are shown for two-way ANOVA in strawberry SA levels.
