# Supplementary material for: The different interactions of Colletotrichum gloeosporioides with two strawberry varieties and the involvement of salicylic acid
Source: Hortic Res. 2016 Mar 16;3:16007–. doi: 10.1038/hortres.2016.7 (PMC4793257; doi:10.1038/hortres.2016.7)

**Journal name**

Horticulture Research

**Article title**

The different interactions of *Colletotrichum gloeosporioides* with two strawberry varieties and the involvement of salicylic acid

Qing-Yu Zhang • Li-Qing Zhang • Li-Li Song • Ke Duan • Na Li • Yan-Xiu Wang • Qing-Hua Gao

Qing-Yu Zhang • Li-Qing Zhang • Li-Li Song • Ke Duan • Qing-Hua Gao (
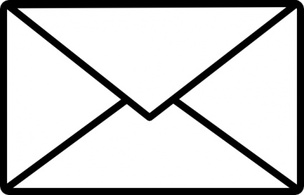
)

*Shanghai Key Laboratory of Protected Horticultural Technology, Forestry and Fruit Tree Research Institute, Shanghai Academy of Agricultural Sciences (SAAS), 1000 Jin-Qi Road, Feng-Xian District, Shanghai 201403, China*

Qing-Yu Zhang

*College of Landscape Architecture and Arts, Northwest A&F University, Yangling, Shaanxi 712100, China*

Li-Li Song • Yan-Xiu Wang

*College of Agricultural Sciences, Gansu Agricultural University, Lanzhou 730000, China*

Na Li

*School of Life Science, Taizhou University, Taizhou 318000, China*

**^*^Corresponding authors:** Qing-Hua Gao

E-mail: [qhgao20338@sina.com](mailto:qhgao20338@sina.com)

**Supplementary files** Two supplementary tables and three supplementary figures are published online.

**Supplementary Table S1** Sequences of the primers used in this study for gene expression analysis.

**Supplementary Table S2** Disease index and leaf incidence in *C. gloeosporioides*-infected strawberry pretreated or not treated with 20 μM SA.

**Supplementary Fig. S1** Typical leaf lesions in whole plants of *Fragaria × ananassa* cvs. Jiuxiang and Sweet Charlie 7 d post inoculation with *C. gloeosporioides*.

**Supplementary Fig. S2** qRT-PCR analysis of dynamic *NBS25* transcript levels in strawberry cvs. JX and SW after spraying with SA at different concentrations.

**Supplementary Fig. S3** qRT-PCR analysis of dynamic *NB-LRR*s transcript levels in strawberry cvs. JX and SW after 20μM SA treatment.

**Supplementary Table S1** Sequences of the primers used in this study for gene expression analysis.

| Gene  Symbol | *F．vesca*  Orthologue | Forward primer (5’-3’) | Reverse primer (5’-3’) |
| --- | --- | --- | --- |
| *FaPR1* | gene01774 | TAGCAGCCTATGCACAAAG | ACAGGTTCACAGCAGATG |
| *FaPR1a* | gene01729 | TGGACAATACGGTGAAAATC | CATAGTTGCACCCAATGAAG |
| *FaPR3* | gene17204 | AACGGTGGACTTGAATGTGG | TCGAGGACACTTTTGATGAG |
| *FaPR5* | gene09812 | CCTAATGACACTCCCGAAACA | AGGTCCACCGAAGCATGTAA |
| *FaPR10* | gene07082 | GAGTTCACCTCAGTCATC | GGTTCCAACACCTCCATC |
| *FaNBS4* | gene07385 | GCTCCCAGTGACTTTCTCC | TCAAAGCCCCCAAACCAA |
| *FaNBS7* | gene13372 | TCAGGAATTATGGAGGACC | TGGAAGACAAACAAGGCT |
| *FaNBS14* | gene16021 | CAGAGTAAGGCGACAAGTGC | CAGAAAGCCAAGAGACGGGTGA |
| *FaNBS17* | gene16021 | TGAATCTGAGTGGGTGTGAG | ACCGACTGAGAAGGGAGGCA |
| *FaNBS21* | gene09518 | TCTTTTCCTTCAGCAAC | GCAGCCACTTAGATTCAG |
| *FaNBS25* | gene00747 | GGCATAATGTTACACTTGGG | GGATGAATCTTAACTAGCTTGG |
| *FaNBS30* | gene12913 | GGAAGTGAAATTCCTGAGTG | CTGAAAACCTGGTGGGAG |
| *FaNBS33* | gene26568 | GGGAACTGAAGAGACTGAAG | TCCGATGGACTGGTCAAT |
| *FaRIB413* | gene33863 | ACCGTTGATTCGCACAATTGGTCATCG | TACTGCGGGTCGGCAATCGGACG |

**Supplementary Table S2** Disease index and leaf incidence in *Colletotrichum gloeosporioides*-infected strawberry pretreated with or not with 20μM salicylic acid (SA).

| Variety | | Jiuxiang (JX) | | Sweet Charlie (SW) | |
| --- | --- | --- | --- | --- | --- |
| Treatment | | No SA | 20 μM SA | No SA | 20 μM SA |
| Disease  Index | 2 DPI | 6.63±0.42 | 4.36±0.24* | 1.87±0.28** | 1.62±0.19 |
|  | 4 DPI | 19.30±1.70 | 14.28±1.68* | 13.17±1.05* | 7.05±0.76** |
|  | 7DPI | 31.37±4.03 | 22.94±4.03* | 19.98±2.99* | 15.33±2.38 |
|  | 10DPI | 55.19±7.40 | 42.62±6.30 | 47.15±7.00 | 24.48±4.48* |
| Leaf  Incidence  (%) | 2 DPI | 33.93±4.17 | 23.16±2.82** | 9.80±1.96** | 8.67±1.33 |
|  | 4 DPI | 55.36±5.95 | 46.33±5.08* | 33.33±3.27** | 30.00±3.33 |
|  | 7DPI | 72.02±11.90 | 62.15±10.73 | 50.98±9.80 | 44.67±8.67 |
|  | 10DPI | 86.31±15.48 | 80.79±13.56 | 83.66±14.38 | 60.00±10.67 |

*Significant differences for SA treatment within variety were indicated on the lanes of SA treated, and the differences between cultivars were indicated on the lanes of no SA under cv. SW (T-test, ** for *P* value < 0.01, * for *P* value < 0.05). Disease index was calculated based on 1-7 rating scales applied to all leaves on each plant (see text). Leaf incidence (%) was calculated as described in text. DPI: days post inoculation. Ten plants were used for each treatment for every experiment. Error bars represent the standard error for three repeats of the experiments.

**Supplementary Fig. S1** Typical leaf lesion symptoms in whole plants of *Fragaria × ananassa* cvs. Jiuxiang (JX) and Sweet Charlie (SW) 7 days after inoculation with *Colletotrichum gloeosporioides*. Upper panels: sole *C. gloeosporioides* inoculation; lower panels: Salicylic acid (SA) pretreatment (20 μM SA, foliar spraying at 4 days prior to *C. gloeosporioides* inoculation). The pots were of the same size (inner diameter, 13 cm).


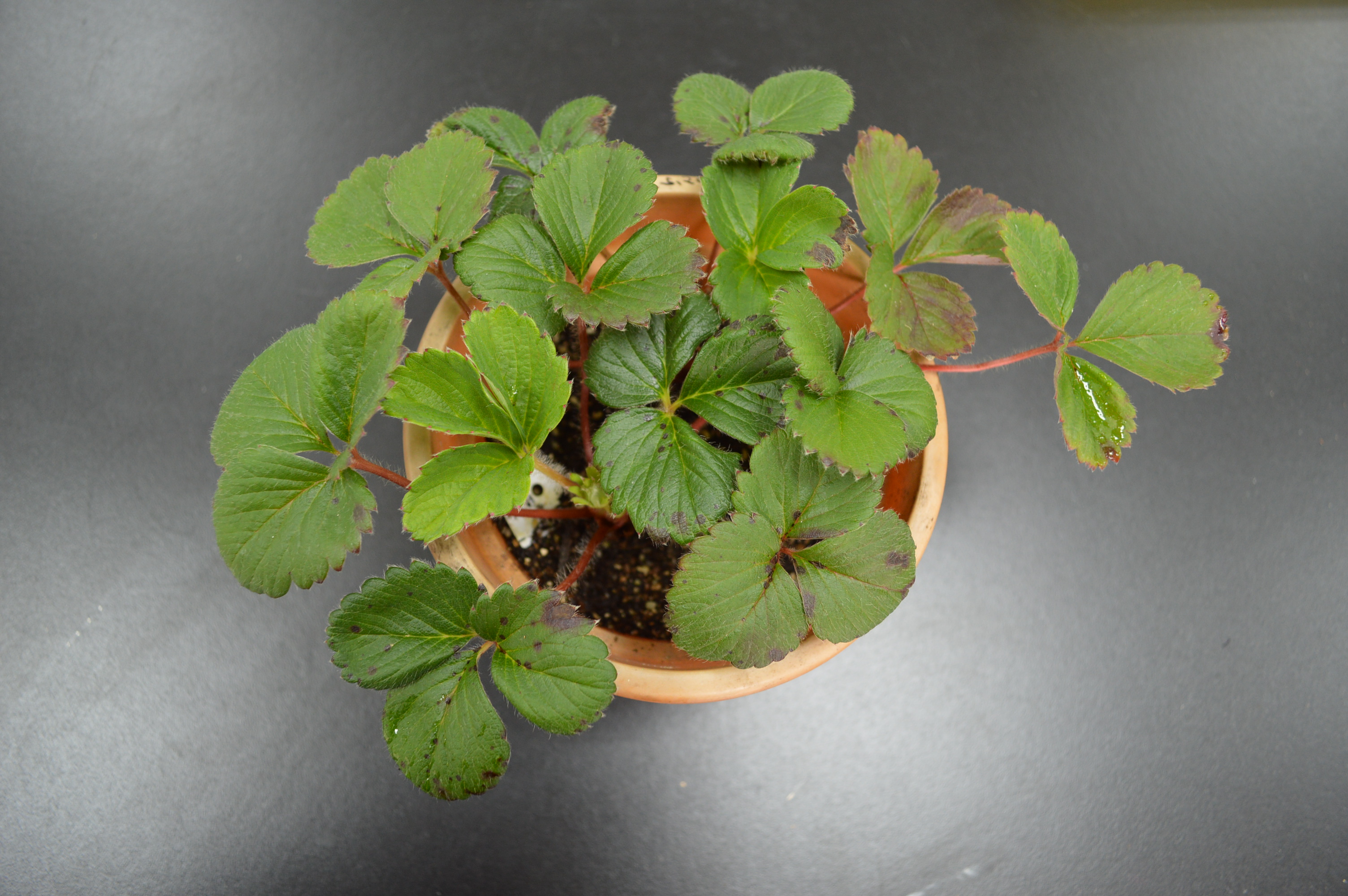

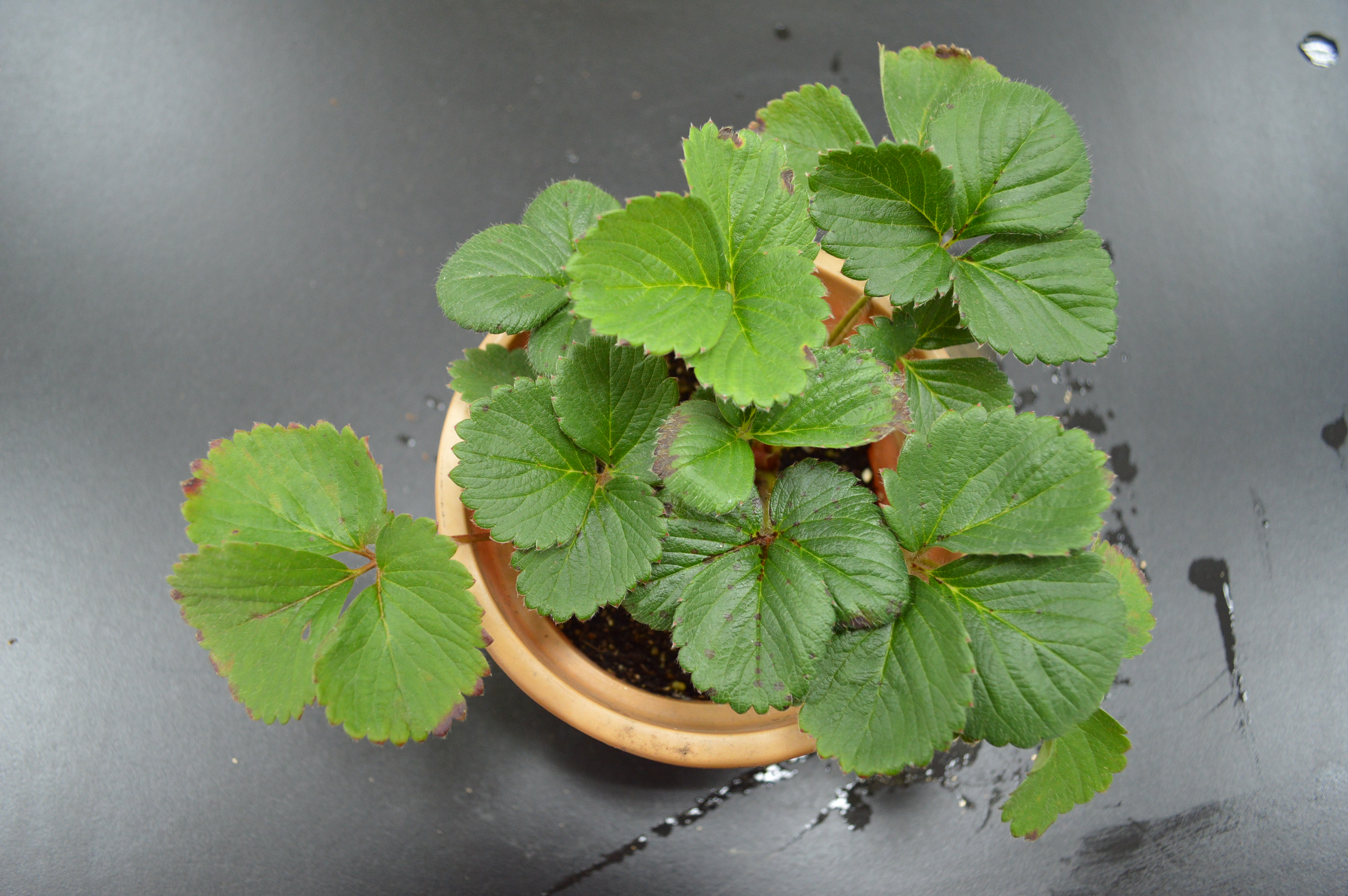

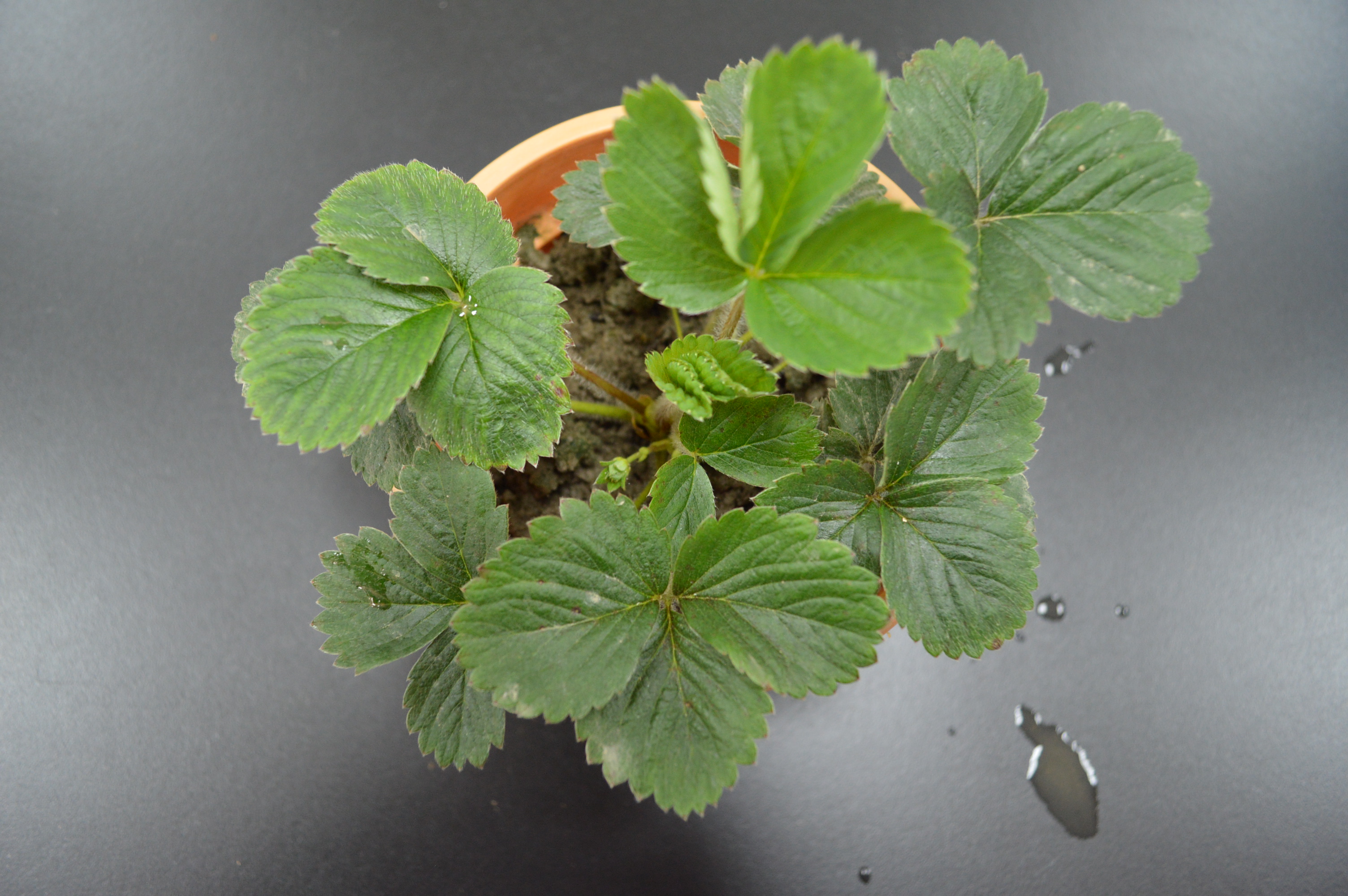


**JX SW**


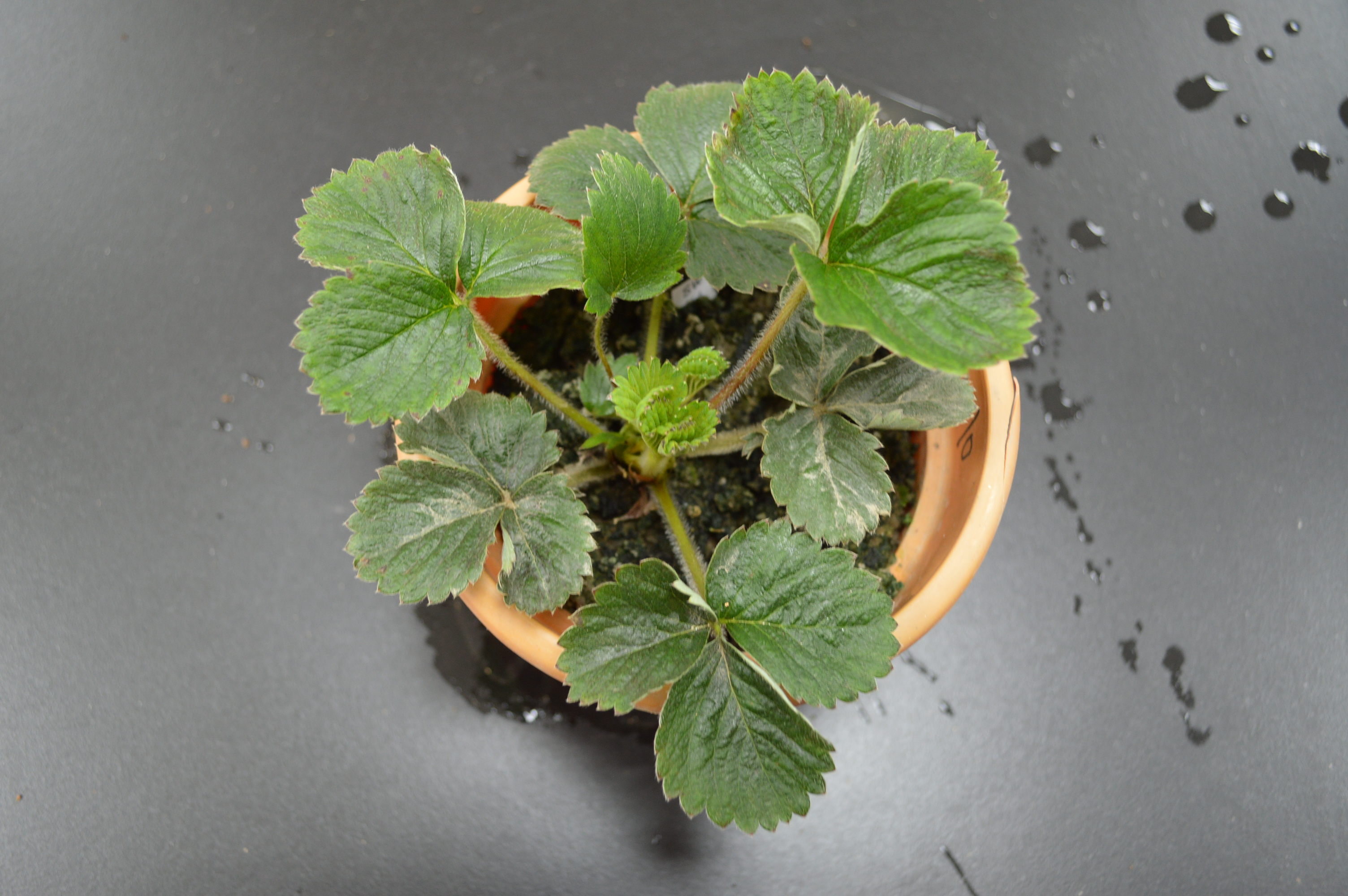


***C.g***

**SA**

**&**

***C.g***

**Supplementary Fig. S2** qRT-PCR analysis of dynamic *FaNBS25* transcript levels in strawberry cvs. JX and SW after spraying with SA at different concentrations. The expression level of each gene in every cultivar was expressed as the fold changes relative to that before treatment (0 H). *FaRIB413* was used for normalization. Bars represent the mean of two independent biological samples ±SE.


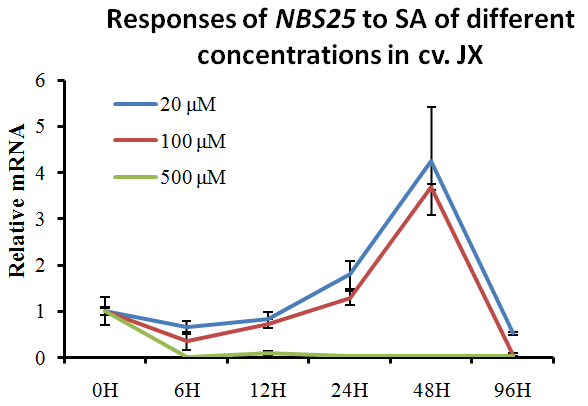

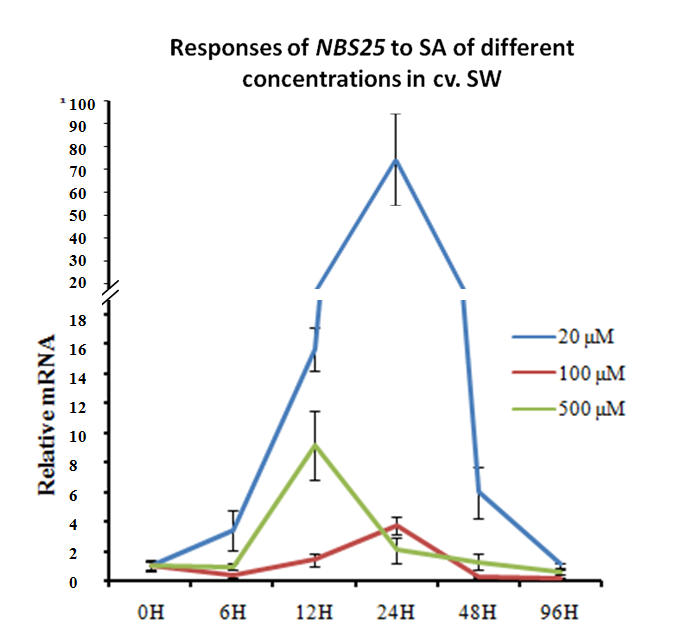


**Supplementary Fig. S3** qRT-PCR analysis of dynamic *NB-LRR*s transcript levels in strawberry cvs. JX and SW after 20μM SA treatment. The expression level of each gene in every cultivar was expressed as the fold changes relative to that before treatment (0 H). *FaRIB413* was used for normalization. Bars represent the mean of two independent biological samples ±SE.


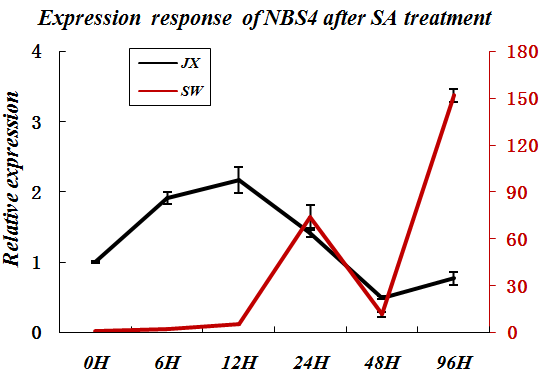

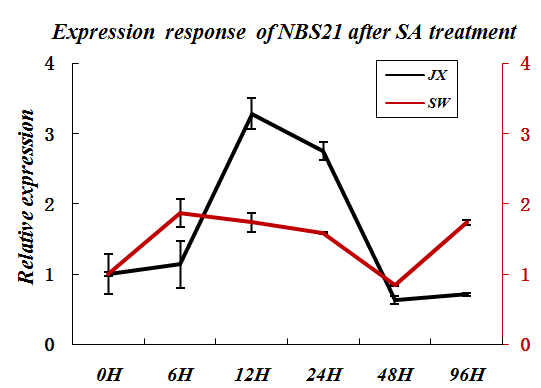

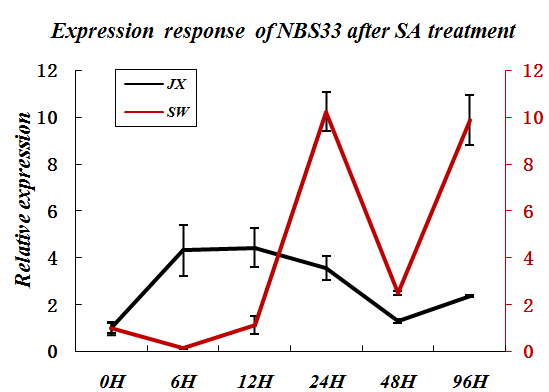

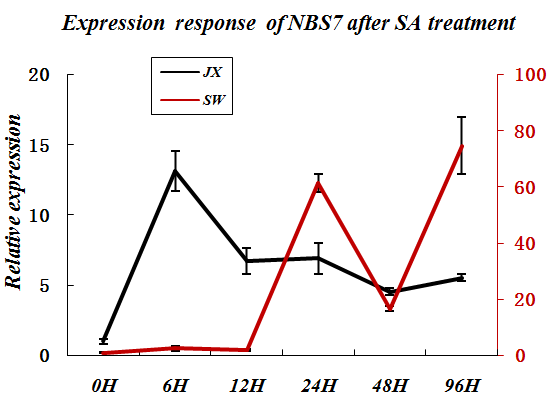

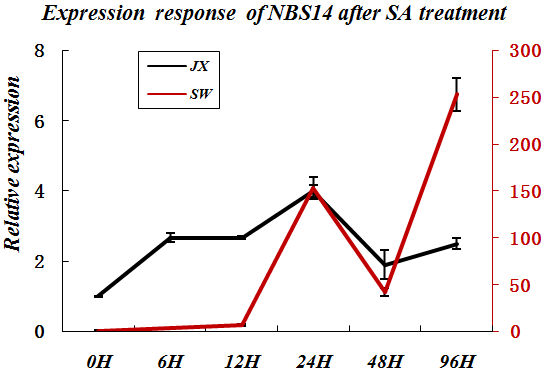

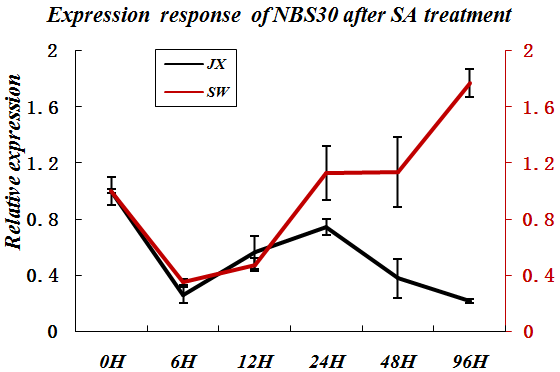

Supplement: Supplementary Information [file hortres20167-s1.doc]
